# Interplay between unfolded protein response and autophagy promotes tumor drug resistance

**DOI:** 10.3892/ol.2015.3508

**Published:** 2015-07-17

**Authors:** MING-MING YAN, JIANG-DONG NI, DEYE SONG, MULIANG DING, JUN HUANG

**Affiliations:** Department of Orthopedic Surgery, Second Xiangya Hospital of Central South University, Changsha, Hunan 410000, P.R. China

**Keywords:** unfolded protein response, autophagy, interplay, tumor, resistance

## Abstract

The endoplasmic reticulum (ER) is involved in the quality control of secreted protein via promoting the correct folding of nascent protein and mediating the degradation of unfolded or misfolded protein, namely ER-associated degradation. When the unfolded or misfolded proteins are abundant, the unfolded protein response (UPR) is elicited, an adaptive signaling cascade from the ER to the nucleus, which restores the homeostatic functions of the ER. Autophagy is a conserved catabolic process where cellular long-lived proteins and damaged organelles are engulfed and degraded for recycling to maintain homeostasis. The UPR and autophagy occur simultaneously and are involved in pathological processes, including tumorigenesis, chemoresistance of malignancies and neurodegeneration. Accumulative data has indicated that the UPR may induce autophagy and that autophagy is able to alleviate the UPR. However, the detailed mechanism of interplay between autophagy and UPR remains to be fully understood. The present review aimed to depict the core pathways of the two processes and to elucidate how autophagy and UPR are regulated. Moreover, the review also discusses the molecular mechanism of crosstalk between the UPR and autophagy and their roles in malignant survival and drug resistance.

## 1. Introduction

When cells face external adverse stress, including glucose depletion, hypoxia and cytotoxicity agents, they respond to these stresses by adaptive autophagy and endoplasmic reticulum (ER) stress to restore homeostasis. Recently, two cell biology processes, autophagy and ER stress, have received extensive attention. A mounting body of knowledge has demonstrated that ER stress and autophagy are central to determining cell fate (1–3). Although the two processes have both been implicated in various human diseases (4–6), the crosstalk between autophagy and the unfolding protein response (UPR) remains poorly understood. Recently, autophagy and UPR have been implicated in malignant cell survival and drug resistance (7–9). The present review focusses on the detailed molecular mechanism of the interplay between ER stress and autophagy. In addition, the effects of ER stress-mediated autophagy on tumor survival and drug resistance are also presented.

## 2. The autophagy pathway and its regulators

Autophagy is a conserved pathway involving lysosomal degradation of long-lived proteins or damaged organelles such as mitochondria and endoplasmic reticulum. Autophagy may promote cell viability or contribute to cell death, depending on the cell types and contexts (10). Autophagy is composed of macroautophagy, chaperone-mediated autophagy and microautophagy (11,12). Microautophagy involves engulfing disrupted cellular organelles directly for lysosomal degradation. In chaperone-mediated autophagy, the cargoes are recognized and marked by chaperone peptide HSC70 and the substrates are translocated to lysosomes or endosomes. In macroautophagy, ubiqiuitinated proteins are identified via adaptors such as p62, NBR1 (neighbor of BRCA1 gene 1), HDAC6 (Histone Deacetylase 6) and Afly (11). Macroautophagy is characterized with formation of double-membraned vesicles to sequester its cargoes, namely autophagosome. Subsequently, the autophagosomes fuse with lysosomes or endosomes where the cargoes are degraded for recycling (13). The detailed mechanism underlying the process of macroautophagy and its role in tumor progression and growth have been investigated in recent years. As the process of macroautophagy has been studied in the most detail, the present review refers to macroautophagy as autophagy and predominantly discusses macroautophagy.

In yeast, autophagy is orchestrated by a series of evolutionarily-conserved autophagy-related genes (ATGs), of which there are >30 (14). The ATGs make a significant impact on different stages of autophagy, including autophagy induction, vesicle nucleation, autophagosomal elongation and eventual maturation (14). Mammalian orthologues of the ATGs have also been discovered. The ULK/ATG13/FIP200 complex is required for the induction of autophagy in osteosarcoma and NIH3T3 cells (15,16). This complex is regulated by mammalian target of rapamycin complex 1 (mTORC1). mTORC1 inhibits the ULK/ATG13/FIP200 complex by phosphorylation of ULK1/2 and ATG13, which suppresses the phoshorylation of FIR2000 and activity of ULK1/2-ATG13-FIP200 complex. Under starvation stress, adenosine 5′-monophosphate activated protein kinase (AMPK) senses the low level of glucose or the low adenosine-triphosphate (ATP)/adenosine monophosphate (AMP) ratio. Activated AMPK then phophorylates and disrupts the TSC1 (tuberous sclerosis complex 1)/TSC2 (tuberous sclerosis complex 2) complex, leading to blockage of mTORC1 and autophagy induction (17). Class III phosphatidylinositol 3-kinase (vps34) combines with beclin-1, p150 and ATG14L to form the class III PI3PK complex (PI3KC3), which generates phosphatidylinositol 3-phosphate to allow recruitment of LC3 and further formation of the autophagosome (18). PI3KC3 has two counterparts, UV radiation resistance-associated genes (UVRAG) complex and rubicon complex. The UVRAG complex consists of Vps34, beclin-1, UVRAG and p150, which contributes to autophagosome maturation (19). By contrast, the rubicon complex is composed of Vps34, beclin-1, rubicon and p150 and disrupts autophagosome maturation (20). This procedure is also suppressed by pharmacological inhibitors of PI3K complex such as 3-methyladenine (3-MA), LY 294002 and wortmanin. In addition, anti-apoptotic proteins such as B-cell lymphoma-2 (Bcl-2) interact with beclin1 and inhibit the nucleation of autophagosome. *c*-Jun N-terminal protein kinase 1 (JNK1) and death associated protein kinase (DAPK) phosphorylate Bcl-2 and positively regulate autophagy (21,22).

**Figure 1.**
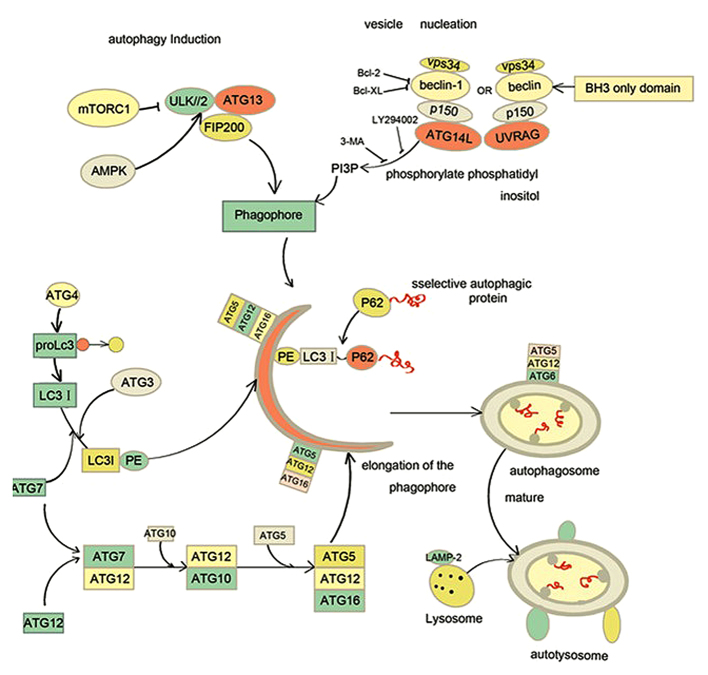
Schematic diagram of molecular events in autophagy pathway. Autophagy is composed of initiation, vesicle nucleation, elongation, maturation, fusion with lysosome and lysosomal degradation. mTORC1, mammalian target of rapamycin complex1; AMPK, Adenosine 5′-monophosphate activated protein kinase; PI3KC3, class III PI3PK complex; LC3, light chain 3; 3-MA, 3-methyladenine.

Two ubiquitin-like conjugation systems are essential for autophagosomal elongation: the ATG12-ATG5 conjugation system and the yeastATG8/mammalian microtubules associated protein 1 light chain 3-β (LC3) conjugation system (14,23). In the former system, Atg12 is covalently conjugated to ATG5 and physically interacts with ATG16L, which is mediated by E1-like enzyme ATG7 and E2-like enzyme ATG10 respectively. The ATG12-ATG5-ATG16L complex acts as a platform to enroll the LC3 II to the isolated membrane (24). LC3 is cleaved at the C-terminal region to produce LC3 I by ATG4 and then E1-like enzyme ATG7 and E2-like enzyme ATG3 mediate LC3 I conjugation with phosphatidylentanolmine to generate LC3 II, the autophagic membrane form (14). Subsequently, the selected engulfed cargo is ubiquitinated and identified by autophagic adaptors, p62/SQSTM1 and neighbor of BRCA1 gene 1 (NBR1). P62 and NBR1 have a similar C-terminal Ubiquitin-binding domain to bind to ubiquitinated cargo and the LC3 interacting region interacts with LC3 (25,26). The last machinery of autophagy is the fusion of autophagosome and lysosomes (Fig. 1). This procedure is dependent on the binding of LAMP1/2, transmembrane proteins present in lysosomes. Once an autophagosome fuses with a lyosome, the cargoes within the autophagosome are degraded for recycling or digested by acid hydrolases and cathepsins in the lysosomal lumen (27).

## 3. The core pathway of the unfolding protein response

The endoplasmic reticulum (ER) is a critical cellular organelle for the quality control of secretory proteins: The ER assists with the correct folding of secreted proteins and polypeptide chains and transmembrane proteins (28). It also contributes to lipid biosynthesis and serves as a site for intracellular Ca2+ storage (29,30). Nascent polypeptide is delivered into the ER lumen and undergoes the correct posttranslational modifications and folding in order to perform their functions efficiently (31). When the protein quality control procedure is well orchestrated, the correctly folded secretory proteins or transmembrane proteins are transferred away from the ER to intracellular apartments or extracellular sites to execute their roles. However, when misfolded or unfolded proteins are in abundance, the accumulation of these proteins triggers ER stress and the UPR ensues (32). The UPR involves an increase in protein folding capacity as well as reducing the unfolded protein load in the ER (33). The UPR triggers a series of cellular processes to recover ER homeostasis: i) The PERK (protein kinase RNA-like kinase) pathway attenuates the load of nascent protein in the ER via the global suppression of protein translation (34); ii) activating transcription factor 6 (ATF6) and inositol-requiring enzyme 1 (IRE1) together improve the protein folding capacity of the ER by upregulating the expression of chaperones and foldase enzymes, which are essential for protein folding (35–37); and iii) IRE1 signaling facilitates ER associated degradation (ERAD) to degrade misfolded proteins and regulate IRE1-dependent decay of mRNA (RIDD) to cleave RNA essential for ER homeostasis (37,38). If the UPR fails to restore ER homeostasis, prolonged and severe ER stress may transform the adaptive UPR response, which protects cells from death in adverse stress, to deadly output by activating the ER-dependent apoptosis signaling pathway (36,39–41).

**Figure 2.**
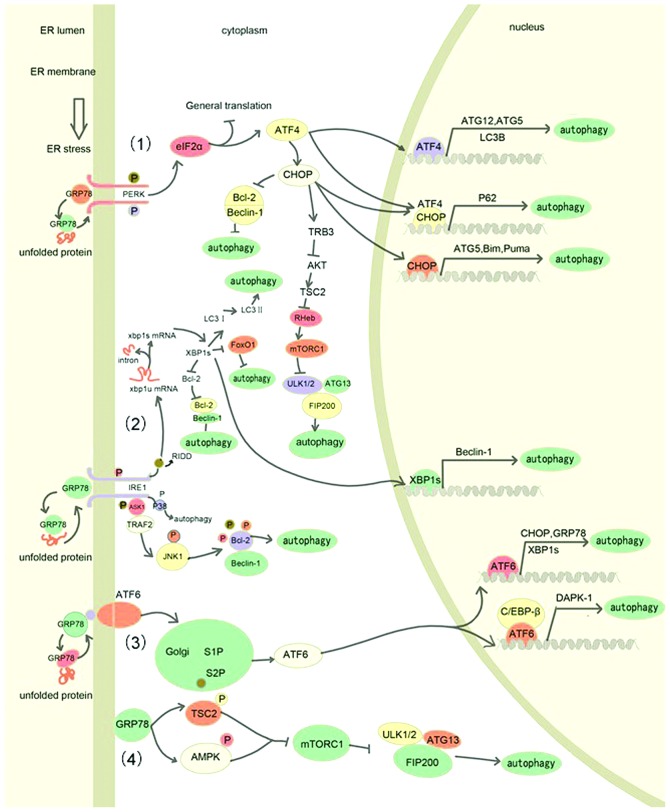
The core UPR pathway and its modulation on autophagy. (1) Signaling from PERK/eIF2α/ATF4/CHOP pathway to autophagy. (2) Signaling from IRE1/JNK1 and IRE1/XBP1s to autophagy. (3) Signaling from ATF6 to autophagy. (4) Signaling from GRP78 and p38 to autophagy induction. UPR, unfolded protein response; GRP78, glucose regulate protein 78; PERK, protein kinase RNA-like kinase; IRE1, inositol-requiring enzyme 1; ATF6, activated transcriptional factor 6; eIF2α, eukaryotic translation initial factor 2α; ATF4, activated transcriptional factor 4; Bcl-2, B-cell lymphoma-2; TRB3, tribbles-related protein3; JNK1, c-Jun N-terminal kinase 1; XBP1s, X-box-binding protein-1 spliced; DAPK1, Death associated protein kinase; TSC2, tuberous sclerosis complex 2; Akt, protein kinase B; FoxO1, Forkhead box O1. ASK1, apoptosis-signal regulating kinase 1; TRAF2, adaptor protein tumor necrosis factor receptor-associated factor-2.

In unstressed conditions, the three transmembrane proteins (PERK, ATF6 and IRE10) are maintained in an inactive state by being sequestrated to glucose regulated protein 78 (GRP78), a light weight chemical chaperone that facilitates protein folding (Fig. 2). During the UPR, the increasing concentration of unfolded protein in the ER lumen completely binds to GRP78 and results in PERK, ATF6 and IRE1 dissociation from GRP78, releasing its inhibitory role on the three sensors. Subsequently, the three UPR sensors are activated and initiate adaptive signal transduction through homodimerization and autophosphorylation (42,43).

## 4. Interplay between UPR and autophagy

That autophagy and ER stress are implicated in cell fate determination has drawn particular attention (2,8,42). However, the detailed molecular mechanism for interplay between autophagy and ER stress remains elusive. In the next section the crosstalk between autophagy and ER stress is discussed.

### 

#### Interplay between IRE1/XBP1s and IRE1/TRAF2/ASK1/JNK with autophagy: IRE1/XBP1s pathway

IRE1 possesses a serine/threonine kinase domain and an endoribonuclease domain (43). In mammalian cells, IRE1 predominantly exerts its pro-survival role through two downstream signaling pathways, namely unconventional splicing of the X-box-binding protein-1 (XBP1) and RIDD (36,38,44). Once IRE1 activated, a 26-nucleotide intron from the mRNA of XBP-1 is unconventionally spliced by the endoribonuclease activity of IRE1 to produce a potent transcriptional factor, spliced XBP-1 (XBP1s) (45). XBP1s contains an activated DNA-binding domain and is a transcriptional factor, which belongs to the basic region/leucine zipper (bZIP) family. Following translocation to the nucleus, XBP1s in turn binds and upregulates the UPR-targeted genes that facilitate the capacity of protein folding, such as GRP78 (46). The IRE1-XBP1s axis has been identified to induce autophagy in different phases. Firstly, XBP1s has been confirmed to induce autophagy indirectly through regulating the expression of Bcl-2 (21,47). In addition, the induction of autophagy is also observed in endothelial cells that overexpress XBP1s, accompanied with increased conversion of LC3 II to LC3II and enhanced expression of beclin-1 (48). Furthermore, it was demonstrated that XBP1s formed a homodimer or heterodimer and directly bound to the −537 and −755 region of the *BECN1* gene promoter, enhancing the expression of beclin-1 (Fig. 2). Although IRE1/XBP1s have a positive regulatory effect on autophagy and elicits a pro-survival signal in the majority of contexts, a deficiency of IRE1/XBP1s results in enhancement of autophagy and viability in cells from amyotrophic lateral sclerosis patients (49). Additionally, XBP1s deficiency results in the elevated expression of Forkhead box O1, a transcriptional factor that promotes autophagy in neurons (50). At present, the paradoxical effect of the IRE1/XBP1s pathway on the induction of autophagy may be attributed to the limited knowledge of this phenomenon.

#### IRE1/JNK pathway

JNK belongs to the mitogen-activated protein kinase (MAPK) super family, which is involved in numerous processes and has been identified as a ‘stress-associated protein’ (51,52). Upon initiation of the UPR, IRE1 is activated and recruits the adaptor protein tumor necrosis factor receptor-associated factor-2 (TRAF2) to form the IRE1-TRAF2 complex (53). Subsequently, apoptosis-signal regulating kinase 1 (ASK1) is enrolled to generate the IRE1-TRAF2-ASK1 complex (54). In the cytosolic facet of ER membrane, JNK is phosphorylated by the serine/threonine kinase domain of IRE1. It has been demonstrated that ER stress inducers such as tunicamycin and thapsigargin can trigger the formation of autophagic vacuoles and accumulation of LC3-positive vesicles in mouse embryonic fibroblast cells (MEFs). ER stress-induced autophagy is dependent on the IRE1/TRAF-2/JNK1 pathway; this is supported by the observation that MEFs that are deficient in IRE1/TRAF-2 display markedly reduced formation of autophagosomes (55). Moreover, SP600125, a pharmacological inhibitor of JNK1, also blocks the formation of autophagosomes (52). Yong *et al* (56) demonstrated that JNK1 contributed to starvation-induced autophagy via phosphorylating ER localized Bcl-2 at multi-residues T69, S70 and S87A, leading to beclin-1 dissociating from ER-localized Bcl-2 and initiating autophagy (56). In addition, the JNK1 pathway was demonstrated to serve a pivotal role in regulating beclin-1 expression at the transcriptional level following ceramide-induced autophagy in human CNE2 and Hep3B cancer lines (52). Ceramide-induced upregulation of beclin-1 and the formation of autophagosomes was inhibited by SP600125, a specific JNK1 inhibitor and the same phenomenon was observed using a small interfering RNA targeting JNK mRNA. Moreover, chromatin immunoprecipitation and luciferase reporter analysis verified that c-jun, a target of JNK1, was activated and directly bound to the beclin-1 promoter in ceramide-treated cancer cells. In this context, the IRE1/JNK1/c-jun pathway is another important mechanism for autophagy induction. It must be noted that the IRE1/XBP1s and IRE1/JNK1 induced-autophagy pathways converge at beclin-1, an ATG protein that is vital during vesicle nucleation. Targeting beclin-1 may be a novel therapeutic strategy to reverse the dysfunction of ER stress-induced autophagy in diseases, including cancer, neurodegenerative disease and diabetes mellitus (57,58).

#### Interplay between the PERK/eukaryotic translation initial factor 2α (eIF2α)/activating transcription factor 4 (ATF4)/CHOP axis and induction of autophagy

Once released from GRP78, PERK, a serine/threonine kinase, is activated through autophosphorylation and homodimerization. The activated PERK phosphorylates eIF2α at serine 51, disrupting the assembly of initiator Met-tRNA and the ribosome, resulting in the suppression of general protein synthesis (59,60). Paradoxically, phosphorylation of eIF2α promotes the translation of ATF4, which enhances the ER's capacity for protein folding (61–63). Subsequently, increased level of ATF4 promotes the translation of CCAAT/enhancer binding protein (C/EBP) homologous protein (CHOP), which acts as a marker to detect the induction of the UPR and is involved in the ER stress-mediated apoptotic pathway (64,65).

It has been demonstrated that polyglutamine (72Q) aggregates induce vesicular formation and conversion of LC3 is dependent on PERK/eIF2α activation. This is supported by the fact that 72Q-mediated induction of autophagy is suppressed significantly in MEF cells with an eIF2αA/A mutation or in MEF cells transfected with PERK (dominant-negative PERK) (66). MEF cells with an eIF2αA/A mutation cannot be phosphorylated by PERK, and dominant-negative PERK MEF cells cannot phosphorylate eIF2αA and can prevent wild-type PERK phosphorylating eIF2αA. In addition, ATF4 is responsible for upregulation of ATG12 (67), a key component of the Atg5-Atg12-Atg16L complex, which is essential for the elongation of autophagosomes. Similarly, BRAF inhibition induces phosphorylation of PERK and is crucial for autophagosome formation in melanoma (68). Blockage of PERK using either the pharmacological inhibitor GSK2606414, or an siRNA against PERK elicits a marked reduction in the LC3 II/I ratio. Furthermore, ATF4 directly binds to the cyclic AMP response element binding site in the promoter of microtubule-associated protein 1 light chain 3 β (LC3β), a vital constituent of autophagosomal membrane, and promotes expression of LC3β, which facilitates the induction of autophagy (67,69).

CHOP is a potent transcription factor, which is implicated in autophagy induction. This transcription factor has been implicated in various cellular processes, including proliferation, differentiation, apoptosis, autophagy and the UPR (70–72). It has been demonstrated that CHOP expression increases the expression levels of ATG5 and BH3-only protein (73,74), in addition to reducing the expression levels of Bcl-2, which contributes to beclin-1 releasing from Bcl-2 (72,75). In addition, CHOP expression also results in increased expression levels of BH3-only proteins such as Bim and Puma, which also bind to Bcl-2 via the single BH3 domain and this further releases beclin-1 from the Bcl-2-beclin-1 complex (74). Furthermore, the PERK-CHOP pathway provokes the induction of tribbles-related protein3 (TRB3), which blocks the activation of protein kinase B (Akt) (76). TRB3-mediated inhibition of Akt attenuates the phosphorylation of TSC2 (tuberous sclerosis complex 2) on serine/theronine residues, leading to an inhibitory regulator of Ras homolog enriched in brain (Rheb) and inactivation of mTORC1. Finally, inactivation of mTORC1 dephosphorylates ATG13 and the ULK1/2 complex and initiates autophagosomal formation (77). The eIF2α/ATF4/CHOP axis promotes the expression of p62 at the transcriptional level through binding to the AARE sequence of p62 promoter to regulate autophagy induction (78).

#### Interplay between ATF6 and autophagy

ATF6 acts as an ER transmembrane sensor, characterized by its C terminus in the ER lumen and N-terminus (possessing the transcription factor activity) in the cytosol (79). When the amount of misfolded protein increases to the threshold, ATF6 escapes from the sequestration of GRP78 and exposes the Golgi localization signals to facilitate its delivery to the Golgi apparatus. ATF6 undergoes cleavage by Golgi apparatus localized site 1 and site 2 proteases (80). Subsequently, the activated ATF6 is translocated to nucleus and then binds to ER stress-associated elements. ATF6 elevates the expression of GRP78, GRP94, XBP1, CHOP and protein disulfide isomerase, which is essential for assisting correct protein folding and secretion (81–83). It has previously been demonstrated that ATF6 is required for induction of autophagy. Death-associated kinase 1 (DAPK1) is involved in ATF6 mediated autophagy (84). The mechanism underlying ATF6 induced autophagy is that ATF6 interacts with C/EBP-β to form a transcriptional heterodimer complex and then binds to CRE/ATF elements of the *DAPK1* promoter to induce the expression of DAPK1. Knockdown of ATF6 with specific shRNAs and cells with ATF6^−/−^ displayed strongly reduced expression of DAPK1 and reduced autophagosome formation. Indeed, DAPK1 has been implicated in driving autophagosome formation through phosphorylation of beclin-1 (85). Meanwhile, ATF6-mediated upregulation of CHOP, XBP1 and GRP78 also contributes to ATF6-induced autophagy (1). This indirect pathway adds a further layer of complexity in ER stress-induced autophagy.

#### Other pathways involved in ER stress-induced autophagy

GRP78 is a key UPR trigger and ER molecular chaperone, which has been demonstrated to induce autophagy. Knockdown of GRP78 suppresses autophagy. However, the siGRP78-dependent autophagy inhibition was reversed following the addition of siXBP-1 (86). Accumulating evidence has demonstrated that autophagy relies on intact ER function and its correct morphology, which provides an essential membrane for autophagosomal elongation and nucleation. Knockdown of GRP78 disrupting normal ER function and morphology may be attributed to the suppression of ER stress-induced autophagy (86). This finding was also strongly supported by the observation that GRP78 induced activation of AMPK and TSC2, which results in the inhibition of mTOR and induction of autophagy in breast cancer (87).

In fibroblasts from patients with Pompe disease, accumulation of misfolded acid α-glucosidase (GAA) induced ER stress and resulted in increased levels of LC3 II and autophagosome formation. Mechanistically, the activation of p38 MAPK signaling pathways were essential for this phenomenon (88). NB-DNJ, a pharmacological chaperone for misfolded GAA, dramatically reduces the level of p38 phosphorylation and p38-associated ER stress. The autophagic flux induced by ER stress was also attenuated following treatment with SB203580, a specific p38 MAPK inhibitor. Similarly, another study uncovered an increase in p38 phosphorylation and induction of autophagy in human gingival cells after exposure to ER stress agents brefeldin A, thapsigargin and tunicamycin (89). In this context, SB203580 suppressed ER stress-induced autophagy. As a downstream target of ER stress, p38 was demonstrated to be phosphorylated by the IRE1/ASK1 axis. Notably, JNK, another MAPK, is known to be a common target of the IRE1/ASK1/TRAF2 pathway. However, the level of phosphorylated JNK and ERK remained unchanged in fibroblasts and human gingival cells in response to ER stress. Therefore, which pathway among the three MAPK pathways is the preferential mediator to induce autophagy in ER stress condition appears elusive at present.

#### Autophagy counterbalances the ER stress

Autophagy alleviated ER stress may also be established, which completes the feedback loop of crosstalk between autophagy and ER stress (90). Normally, ER stress induces a process that delivers misfolded proteins to the cytoplasm where they are ubiquitinated and degraded by the ubiquitin-proteasome system, which is termed ER associated degradation (ERAD) (91). However, when the process of ERAD is saturated or disrupted, ER stress-induced autophagy is considered to degrade the insoluble misfolded or unfolded proteins to alleviate the ER stress and recover homeostasis (92,93). This hypothesis is supported by a study that reported that HCT116 and DU145 cells displayed increased levels of ER stress following impairment of autophagy via a pharmacological inhibitor or the transfection of an siRNA targeting BECLIN-1 or LC3B (92). Furthermore, autophagy may counterbalance ER expansion by sequestering the ER into double membrane-bounded and autophagosomal-like structures (94). Rapamycin, a well-established autophagy inducer, has been observed to reduce hypoxia/ischemia-induced ER stress significantly *in vivo* (90). In addition, 3-methyladenine, an early pharmacological inhibitor of autophagy, completely reverses the inhibition of ER stress (90).

## 5. The role of UPR and autophagy in malignancies, drug resistance and survival

Previous studies have indicated that autophagy and ER stress protect cancer cells exposed to various stresses from death (57,95). Osteosarcoma cells display increased levels of autophagosomal formation when treated with anticancer agents, including cisplatin, doxorubicin and methotrexate (96). The autophagy induced by anticancer agents is suppressed by 3-MA or knockdown of beclin-1, ATG7 or PI3KC3, which may sensitize the osteosarcoma to the anticancer agents. These finding indicate that autophagy in response to anticancer agents may contribute the chemotherapeutic resistance of osteosarcoma (96,97). The latest findings about autophagy and ER stress mediated pro-survival and drug resistance in malignancies are now presented.

### 

#### The role of GPRP78 in cancer survival and drug resistance

An upregulated level of GRP78 has been observed in various cancers (98,99). This canonical chaperone is the major effector that protects cancer cells from death in ER stress conditions. Elevated GRP78 expression has been closely linked to chemotherapy failure (100,101). Downregulation of GRP78 attenuates tumor formation of colon cancer cells *in vivo* and promotes apoptosis of colon cancer cells *in vitro* (102).

**Figure 3.**
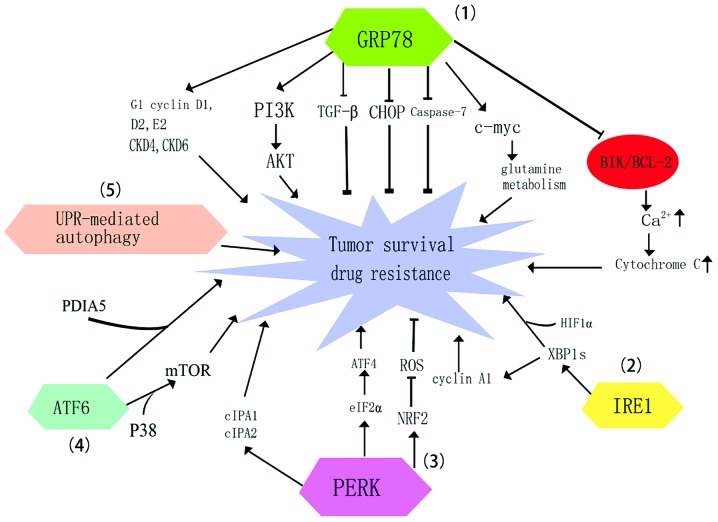
The molecular events of UPR and autophagy induced tumor survival and drug resistance. (1) The role of GPRP78 in cancer survival and drug resistance. (2) The role of IRE1/XBP1 in cancer survival and drug resistance. (3) The role of PERK in cancer survival and drug resistance. (4) The role of ATF6 in cancer drug resistance. (5) The role of ER stress induced autophagy in malignancy drug resistance. CKD, cylin-dependent kinase; PI3K, phosphatidylinositol 3-kinase; ROS, reactive oxygen species; TGF-β, transforming growth fator-β; HIF1α, hypoxia inducible factor-1; cIPA1, cellular inhibitor of apoptosis 1; cIPA2, cellular inhibitor of apoptosis 2; NRF2, nuclear factor erythroid-2-related factor 2; PDIA5, protein disulfide isomerase 5.

The molecular mechanism for GRP78 protecting tumor cells against chemotherapeutic agents as established from previous studies is as follows (Fig. 3): i) GRP78 binds and inactivates the pro-apoptotic protein caspase-7, which is localized in the ER outer membrane (103,104). GRP78 simultaneously mediates CHOP suppression and reduces CHOP-dependent apoptosis. ii) It has been proposed that GRP78 may bind to BIK through its BH-3 domain, which reverses the increased BIK expression in breast cancer that results from the presence of anti-estrogen agents, disrupting BIK/BCL-2 complex formation. An increase of free BCL-2 in the ER membrane then alleviates Ca^2+^ leakage, in addition to the release of cytochrome c from mitochondria to the cytoplasm, blocking caspase-dependent apoptosis (104,105). iii) Silencing GRP78 inhibits renal cell carcinoma (RCC) growth and induces G1 cell-cycle arrest. Knockdown of GRP78-induced activation of the UPR results in a marked suppression of G1/S translation-associated cyclins (D1, D2 and E2) and cylin-dependent kinase (CKD4 and CKD6) expression (101). iv) A study has demonstrated that GRP78 may be delivered to and anchored at the cell surface as a receptor during ER stress (106). The cell surface GRP78 activates the PI3K/AKT pathway and interacts with Cripto to suppress the transforming growth factor-β (TGF-β) pathway, promoting cell survival and growth (10). This data is supported by a number of previous studies that have demonstrated that upregulation of GRP78 results in the chemoresistance phenotype of breast cancer, malignant gliomas and tumor associated endothelia cells (100,107,108).

The characteristics of cancer cells include high rates of proliferation, insufficient supply of oxygen and glucose, apoptotic resistance and angiogenesis; therefore, cancer cells require increased rates of aerobic glycolysis and glutamine consumption for growth (109). *c*-Myc-dependent glutamine metabolism has been implicated in assisting cancer cell survival during glucose deprivation (110). It has been indicated that elevated levels of GRP78 induce *c*-Myc expression and promote *c*-Myc-mediated glutamine metabolism, which contributes to cell survival (111). The mechanism for enhanced c-Myc-mediated glutamine metabolism is attributed to GRP78 disrupting adenomatous polyposis coli (APC)-β-catenin and E-cadherin β-catenin complexes, which results in the extracellular release of APC and an enhanced level of free β-catenin. Eventually, the high expression levels of intermediaries of the β-catenin pathway facilitates the c-Myc-mediated glutamine metabolism. This finding adds support to the hypothesis that GPR78 acts as a novel link between metabolic changes and tumor survival. Taken together, these observation indicate that overexpression of GRP78 occurs in tumors and confers drug resistance (112). Targeting GRP78 may be a novel strategy to overcome the barrier of chemotherapeutic failure in the future.

#### The role of IRE1/XBP1 in cancer survival and drug resistance

Under most circumstances, the UPR is considered to be a cytoprotective response, whose main goal is to reduce the protein load that requires folding and to increase the capacity for folding protein in the ER lumen (40). In the three major branches of UPR, the IRE1 is considered to elicit the pro-survival output. IRE1 has been demonstrated to be associated with cancer proliferation and angiogenesis *in vitro* and *in vivo* (113). Glioma cells that express dominant-negative IRE1α display a markedly reduced growth rate and reduced angiogenic signalling (114). In addition, persistent activation of IRE1 was also responsible for the resistance of melanoma cells to ER stress-induced apoptosis (115).

XBP1s and RIDD are two potent IRE1-induced pro-survival signals that occur in adverse stress (36). IRE1 mediated unconventional splicing of XBP-1 mRNA and regulation of cyclin A1 expression favors IRE1-induced cancer cell growth (Fig. 3) (116). Elevated levels of XBP1 splicing have been observed in various types of tumor and predict poor outcomes for patients (117,118). Triple-negative breast cancer (TNBC) is an aggressive tumor with few effective treatment options characterized by the absence of estrogen receptor, progesterone receptor and HER2 (human epidermal growth factor receptor-2) expression but high levels of XBP1s expression. Impairment of XBP1 splicing markedly inhibits TNBC growth, metastasis and angiogenesis (119). In addition, xenograft mice transfected with MDA-MB-231 breast cancer cells with an shRNA targeting XBP1, reduces the risk of breast cancer tumor relapse (119). Furthermore, it has been established that XBP1s binds to hypoxia inducible factor-1α (HIF1α) via its amino-terminus-bZIP domain and promotes the expression of HIFα targeting genes, including vascular endothelial growth factor-A, phosphoinositide-dependent kinase 1, GLUT1 and DNA-damage-inducible transcript 4, which confer pro-survival signaling responses to hypoxic stress (119). Similarly, another study indicated that XBP1s is critical in myeloma pathogenesis and a high ratio of XBP1s/XBP1 unspliced is closely correlated with poor outcome and a shortened relapse interval in patients (120). It has also been identified that the blockage of IRE1α endoribonuclease activity with novel small molecules such as MKC-3946 and STF-083010, inhibits the splicing of XBP1 in multiple myeloma (MM) when in untreated condition. In addition, MKC-3946 treatment also leads to a significant suppression of XBP1 splicing and enhancement of ER mediated apoptosis in MM cells when concurrently treated with bortezomib or 17-allylamino-17-demethoxygeldanamycin (17-AGG). Treatment with either of the two specific IRE1 endoribonuclease inhibitors exerts no effect on the kinase activity and autophoshorylation of IRE1 but only marked inhibition of XBP1 splicing and its downstream substrates, which strongly demonstrates that the IRE1-XBP1 axis is essential for MM cell survival and targeting this pathway may result in marked anti-tumor effects (120).

#### The role of PERK in cancer survival and drug resistance

Dimerization and autophophorylation of PERK ensues following dissociation from GRP78; PERK then phosphorylates eIF2α at serine 51 and nuclear factor erythroid-2-related factor 2 (NRF2) (121). Activation of NRF2 promotes resistance to hypoxia in cells through enhancing the expression of enzymes that scavenge reactive oxygen species (ROS) (122). Knockdown of PERK sensitizes esophageal and breast tumor cells to chemotherapeutic agents and impairs the growth of these two malignant types of cancer *in vitro*, which is attributed to the activation of the double stranded DNA breakage checkpoint to trigger G2/M arrest and the accumulation of ROS (Fig. 3) (123). A highly selective PERK inhibitor, GSK2656157, has been demonstrated to block the ER stress-induced PERK autophosphorylation and attenuate the phosphorylation of eIF2α and expression of downstream messengers, ATF4 and CHOP (124). Furthermore, treatment with GSK2656157 robustly reduced angiogenesis and altered amino acid metabolism, which impaired human xenograft tumor growth in mice (124). Similarly, ATF4^−/−^ cells demonstrate increased sensitivity to hypoxic stress (125). Moreover, another novel mechanism underlying PERK-dependent pro-survival signaling has been reported by Hamanaka *et al*. PERK induced upregulation of cellular inhibitor of apoptosis (cIPA1 and cIPA2) contributes to the protection of cells against tunicamycin-induced death (126). Finally, the PERK-eIF2α axis is robustly elevated in chronic myeloid leukemia (CML) cells that also express high levels of BCR-ABL (127). Meanwhile, genetic modification of CML cells via transfection with dominant-negative mutants of PERK or dominant-negative eIF2α-S51A mutant, results in markedly increased levels of apoptosis when treated with imatinib (127). Indeed, compromised PERK-eIF2α phosphorylation significantly extends the population doubling time and results in smaller clone sizes in human K562 CML cells with dominant-negative PERK or eIF2α. Collectively, the PERK arm substantially contributes to the growth of tumor cells and elicits a dominant pro-survival output in tumor cells when treated with anti-tumor agents (127). Thus, targeting the PERK-eIF2α pathway represents another promising strategy to override the barriers for dealing with malignant tumors.

#### The role of ATF6 in cancer drug resistance

The role of ATF6 in tumor chemoresistance has not been extensively studied at present. However, accumulating evidence on ATF6-dependent tumor drug resistance has uncovered that ATF6 is another contributor to cancer drug resistance. The detailed mechanism for ATF6 activation and ATF6 induced imatinib resistance in leukemia has been described (128). In this model, protein disulfide isomerase 5 (PDIA5) was identified as being essential for ATF6 activation and export of proteins from the ER lumen. Ablation of PDIA5 reduced the expression of ATF6 specific target genes. Furthermore, silencing of ATF6 expression sensitized K562R cells (a leukemia cell line resistance to imatinib) to the treatment of imatinib. In addition, persistent activation of ATF6 and reduced apoptosis were revealed in tunicamycin or thapsigargin-treated melanoma. It may therefore be concluded that ATF6 activation is essential for protecting melanoma against ER stress-induced cell death (115). In addition to the roles of ATF6 in cell survival and drug resistance in proliferating malignant tumor cells, ATF6 mediated pro-survival and chemoresistance in dormant tumor cells: ATF6 was demonstrated to be responsible for tumor relapse in the human dormant squamous carcinoma cell line, D-HEp3 (129). P38 signaling dependent activation and nuclear localization of ATF6α has been demonstrated in D-HEp3 by immunoblotting and immunofluorescence analysis. Moreover, when D-HEp3 cells in which ATF6α expression has been knocked down are treated with doxorubicin, the number of viable cells is markedly reduced (129). The mechanism by which ATF6α elicits its anti-chemotherapeutic effects in D-HEp3 cells is considered to be dependent on the activation of mTOR, supported by the evidence that knockdown of Rheb sensitizes the D-HEp3 to tunicamycin (129).

#### The role of ER stress-induced autophagy in malignancy drug resistance

Depending on the cell types and context, autophagy has been considered to promote tumor survival or facilitate tumor suppression (8,9,130). Mounting evidence has demonstrated that the UPR induces autophagy in various types of malignant tumor (131,132). A number of studies have also strongly indicated that UPR induced autophagy is critical for malignant tumors cells to survive in adverse stress (55,133).

Melanoma exhibits elevated levels of ER stress and autophagy following treatment with the specific BRAF inhibitor, PLX4720. The BRAF inhibitor induced autophagy relies on PERK-dependent ER stress. Blockage of the UPR induced autophagy limits melanoma resistance to the BRAF inhibitor (134). Notably, UPR induced autophagy promotes survival in HCT116 and DU145 cell lines when exposed to ER stress inducers (92). By contrast, suppression of UPR-induced autophagy reduces cell death in primary colon cells and MEF cells when treated with ER stress inducers (92). This observation indicates that UPR induced autophagy exerts a pro-survival response in malignant tumor cells but elicits a cell death response in normal cells. Moreover, another study also strongly supports this hypothesis. The human P493-6B cell line, which expresses high levels of the oncogene c-Myc, exhibits elevated levels of UPR and autophagy (135). This c-Myc induced UPR protects P493-6B cells against c-Myc induced cell death. To further uncover the mechanism of UPR pro-survival consequence, Hart *et al* demonstrated that there is enhanced autophagosome formation and increased LC3 I/II conversion in P493-6B cells with high expression levels of *c*-Myc (135). Meanwhile, PERK ablation attenuates *c*-Myc induced autophagy, indicating the critical role of PERK in autophagy induction. Moreover, disrupting autophagy with bafilomycin A1 in P493-6B cells increases cell death in response to *c*-Myc activation. Therefore, UPR induced autophagy has a pro-survival role in this context.

## 6. Conclusion

The present review describes the core pathway of autophagy and UPR, in addition to their regulations. Moreover, the detailed molecular mechanisms underlying the crosstalk between autophagy and ER stress are discussed. Accumulating evidence identifies that the three arms of UPR exert marked influences on the induction of autophagy. In turn, autophagy also counterbalances ER stress via degradation of protein aggregates and attenuation of ER expansion. This negative feedback loop allows an insight into the intrinsic orchestrated pathways in cells under adverse conditions. In the present review, the roles of UPR induced autophagy in malignant tumor survival and drug resistance were also discussed. Targeting the UPR induced autophagy response may guide novel therapeutic approaches. Given the vital role of the UPR and autophagy in determining tumor cells fate, further studies on how to manipulate these cell processes are essential to broaden our concepts on tumor therapeutic strategies.
